# The Superiority of T2*MRI Over Serum Ferritin in the Evaluation of Secondary Iron Overload in a Chronic Kidney Disease Patient: A Case Report

**DOI:** 10.2147/JBM.S319591

**Published:** 2021-07-26

**Authors:** Abdulrahman Al-Mashdali, Tahiya Alyafei, Mohamed Yassin

**Affiliations:** 1 Department of Internal Medicine, Hamad Medical Corporation, Doha, Qatar; 2 Department of Clinical Imaging, Hamad Medical Corporation, Doha, Qatar; 3 National Center for Cancer Care and Research, Department of Oncology, Hematology and BMT Section, Hamad Medical Corporation, Doha, Qatar

**Keywords:** secondary iron overload, chronic kidney disease, serum ferritin, T2*MRI, liver iron concentration

## Abstract

Secondary iron overload is increasingly encountered in chronic kidney disease (CKD) patients because of the frequent use of parenteral iron products, especially in hemodialysis patients. Serum ferritin has been commonly used to monitor iron overload in these patients; however, other conditions can be associated with the high serum ferritin, like infections and inflammatory conditions. Currently, T2*MRI of the heart and liver is the preferred investigation for evaluating liver iron concentration (LIC) and cardiac iron concentration, which reflect the state of iron overload. Few studies observe a positive correlation between serum iron and LIC in CKD patients and postulate that serum ferritin exceeding 290 mcg/L should indicate significant iron overload and necessitates further MRI evaluation. However, here, we present a patient with a history of ESRD for which she underwent renal transplantation twice referred to our clinic due to persistent elevation in serum ferritin level (>1000 mcg/L) for several years. T2*MRI of the heart and liver revealed the absence of iron overload. Our objective of this case is to demonstrate the accuracy of T2*MRI over serum ferritin in evaluating iron overload and questioning the positive correlation between serum ferritin and LIC in CKD patients.

## Introduction

Our body normally contains up to 4 grams of iron, mainly in hemoglobin.^1^ The liver is considered the main storage site for iron in the body. Total body iron content is established by balancing iron intake from diet or other sources like blood transfusion and iron loss, such as from menstrual bleeding or epithelial cells shedding.^2^ However, no physiological mechanism for the excretion of excess iron has been found in the human body.^3^ Iron overload occurs either due to the excess of iron intake, which exceeds its loss from the body, like thalassemia and sickle cell disease patients requiring chronic blood transfusion^4^,^5^ or due to the increase in iron absorption from the gut despite normal intake, such in hereditary hemochromatosis.^6^ Less frequently, iron overload can happen due to iatrogenic intoxication.^7^

In general, secondary iron overload in ESRD patients is uncommon; however, iron overload has been increasingly diagnosed in the ESRD population over the last decade for multiple reasons. First, given the risk of iron deficiency with erythropoiesis-stimulating agents (ESA) therapy, KDIGO 2012 guideline set 500 µg/L as the upper limit of serum ferritin in ESRD patients on hemodialysis, which encouraged the clinicians to give more intravenous iron to prevent iron deficiency in such patients. Moreover, the recent advance in the diagnostic test for iron overload (particularly MRI) plays a significant role in the early detection of more iron overload cases.^8^,^9^

Because of their availability and lower cost, serum ferritin and transferrin saturation (TSAT) are frequently used to monitor iron accumulation in the body. Though, these parameters can be affected by different conditions, especially infections and variable inflammatory conditions. However, the evidence for the correlation between serum ferritin with liver iron concentration (LIC) and heart iron concentration is still not well established in patients with chronic kidney disease suspected to have iron overload.^10^

Here, we present a patient with a history of end-stage renal disease (ESRD) and renal transplants referred to our clinic because of persistently elevated serum ferritin for several years. T2* MRI of the heart and liver revealed no evidence of iron overload. In this case, we highlight the superiority of T2* MRI of the heart and liver over serum ferritin in evaluating iron overload in CKD patients.

## Case Presentation

A 57-year-old female who referred to our hematology clinic due to persistent elevation in her serum ferritin. Her past medical history was significant for end-stage renal disease (ESRD) due to diabetic nephropathy, for which she underwent kidney transplantation on two occasions. The first kidney transplant was done in 2002 (then, her kidney functions were stable from 2002 until 2016, when they started to deteriorate due to recurrent diabetic nephropathy in the transplanted kidney leading to chronic allograft dysfunction, which required hemodialysis thrice weekly for six months before the second transplant), and the second one in 2017 (Both kidneys transplants were donated voluntarily, the first one from patient’s relative and the second from family’s friend, with written informed consent, and the organ donation was conducted in accordance with the declaration of Istanbul). The patient also had a past medical history of hypertension and breast cancer diagnosed in 2006 treated with a combination of lumpectomy, chemotherapy, and radiotherapy.

At her initial visit to our clinic, the patient denied any symptoms related to iron overload complications. Her vital signs were within the normal limit. Her body mass index (BMI) was 27. Laboratory findings were significant for serum ferritin of 1219.0 μg/L (normal level < 300), serum iron of 14 μmol/L (normal 9–30), TIBC of 50 μmol/L (normal 40–80), iron saturation of 38% (normal 15–45%). Her liver function tests are normal. Also, Her HbA1C levels were between 7% and 8% for the last five years. Of note, her serum ferritin was repeatedly exceeding 1000 μg/L for the last five years (her renal functions, serum ferritin, TSAT were near normal from 2002 until 2016). Our patient did not have any chronic infection, inflammatory condition, liver disease, or active malignancy (she was asymptomatic with normal inflammatory markers) that could lead to elevated ferritin levels over the last five years. The patient denied smoking or alcohol drinking. We summarized the relevant data from 2016 to 2021 in Table 1.

**Table 1:** Summary of Relevant Data from 2016 to 2021 (Since the Detection of the Elevated Serum Ferritin)

Data	Reference Value	2016 (HD Initiated by the End of This Year)	2017(Before Renal Tx)	2018 (After Renal Tx)	2019	2020	2021 (When T2*MRI Done)
Hb level (range)	12–15 gm/dL	10.2–11	8.8–10.4	11.6–12.2	11.3–11.8	10.9–11.4	12.2–12.9
WBC	4–10 x10^3/uL	7.3	6.5	3.9	4.6	6.2	5.5
Serum ferritin	< 300 mcg/L	282	1150	1830	1650	1933	1219
TSAT	15–45%	39	49	52	46	48	38
Creatinine(range)	50–95 umol/L	168–205	320–380 (required HD 3 times /week)	75–118	105–115	100–120	105–123
Iron received	Total	None	Intravenous ferrous carboxymaltose (> 3 gm over 6 months)	None	None	None	None
Blood transfusion	Units/year	None	Two units of PRBC before renal Tx	None	None	None	None
ESA	Weekly	None	Received darbepoetin for six months before renal Tx	None	None	None	None
CRP level	< 6 mg/L	4	11.4	6.4	4.2	3.8	2.1

**Abbreviations:** CRP, C-reactive protein; Hb, hemoglobin; HD, hemodialysis; TSAT, transferrin saturation; TX, transplantation; WBC, white blood cells.

**Figure 1:**
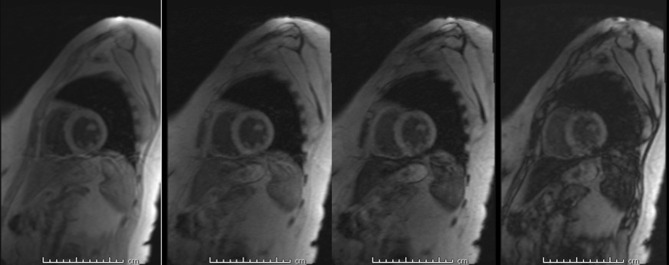
MRI 1.5 T (Siemens Avanto), using multi-TE gradient echo T2* MRI technique. Heart intensity is normal seen with the longest TE (14.68 msec). T2* =29.4 ms corresponding to <1.2 mg Iron/ g heart dry weight.

**Figure 2:**
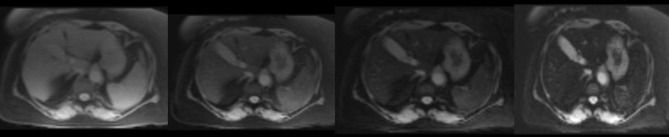
MRI 1.5 T (Siemens Avanto), using multi-TE gradient echo T2* MRI technique (using Garbowski method). Liver intensity is normal seen with the longest TE (14.68 msec). T2* =7.4 ms, corresponding to < 5 mg Iron/ g liver dry weight.

Given her past medical history of ESRD requiring hemodialysis, secondary iron overload was suspected. Accordingly, an T2*MRI of the heart and liver was suggested to rule out iron deposition in body organs. MRI heart revealed iron deposition of less than 1.2 mg/g of dry heart weight. The MRI liver showed an iron deposition of less than 5 mg/g of dry liver weight, consistent with the absence of myocardial and hepatic iron overload (Figures 1 and 2). Table 2 shows the severity classification of iron overload (both for liver and heart) based on T2*MRI findings. Accordingly, the patient was reassured and treated conservatively. In most recent follow-up in our clinic, the patient was doing fine and asymptomatic, and her latest serum ferritin was 940 μg/L.

**Table 2:** Severity of Iron Overload Based on Hepatic and Myocardial T2*MRI and Our Patient Findings

Iron Load Severity	Normal	Mild	Moderate	Severe	Our Patient
Hepatic T2* by millisecond(ms), mg iron/ g of liver dry weight	>7.2ms,< 5 mg/g	3.3–7.2 ms, 5–10 mg/g	2.2–3.3 ms, 10–15 mg/g	< 2.2 ms, > 15 mg/g	T2*=7.4 ms, corresponding to < 5 mg iron/ g of liver dry weight (Normal)
Myocardial T2*by millisecond(ms), mg iron/ g of heart dry weight	> 20 ms,<1.2 mg/g	14–20 ms, 1.2–1.8 mg/g	10–14 ms, 1.8–2.7mg/g	< 10 ms, >2.7mg/g	T2* =29.4 ms, corresponding to <1.2 mg iron/ g of heart dry weight (Normal)

## Discussion

Iron overload is classified into primary and secondary iron overload. The most common cause of the primary iron overload is hereditary hemochromatosis. The secondary iron overload might occur in the context of different hematological disorders, mainly hemoglobinopathies and CKD. Different mechanisms can lead to iron accumulation associated with hematological diseases, including long-term blood transfusion, chronic hemolysis, increased intestinal absorption of iron due to ineffective hematopoiesis, or genetic mutation in hepcidin.^11^,^12^ Iron overload with CKD may happen due to frequent intravenous iron use, especially in patients on hemodialysis. However, the risk of iron toxicity in ESRD patients is usually insignificant because other factors minimize iron accumulation, including the concurrent use of erythropoiesis-stimulating agents (ESA) and the distribution of hepatic iron by the reticuloendothelial cells.^13^ However, as mentioned in the introduction, iron overload is increasingly discovered in hemodialysis patients since that the majority of ESRD patients treated with ESA receive parenteral iron to replenish iron storage and ensure iron availability for hematopoiesis.^8^

Iron overload mainly affects the parenchymal cells of the liver, heart, and endocrine organs. Iron accumulation leads to tissue inflammation and damage through the formation of reactive oxygen species.^14^ As the main store for iron in the body, the liver is the most affected organ by iron overload. Different manifestations may result from excessive iron deposition in the liver, ranging from elevated liver enzymes to liver cirrhosis. Indeed, liver cirrhosis is rarely encountered in hemodialysis patients who developed iron overload.^15^,^16^ Iron deposition in the myocardial tissue can occasionally lead to cardiomyopathy.^17^ Also, iron overload might increase atherosclerosis risk in ESRD patients.^18^ In addition to its effects on different organs, iron overload may increase the risk of infection by disturbing the functions of different immune cells and, as an essential element, by enhancing bacterial growth and multiplication.^16^

Liver iron concentration (LIC) provides a precise representation of body iron storage in patients with iron overload.^19^ At present, MRI becomes the modality of choice for the assessment of iron overload.^20^ The sensitivity and specificity of standardized MRI protocol in determining LIC are estimated to be 94% and 100%, respectively.^21^ The role of MRI methods for evaluating iron overload is well recognized in transfusion-dependent anemia patients; however, this role is still not well established in ESRD patients.^12^,^16^ Given the very high sensitivity (resulting in the low false-negative result) of T2*MRI in the diagnosis of iron overload, we excluded secondary iron overload in our patient, especially in the absence of any clinical or laboratory evidence for that. Few studies were evaluated the correlation between serum ferritin and LIC in CKD patients on hemodialysis by using various MRI standardized protocols. A study done by Canavese et al assumed that the risk of iron overload is ten times more in CKD patients with serum ferritin value surpasses 500 mcg/L.^22^ Another study concluded that there is no correlation between serum iron markers and LIC in CKD patients.^23^ The latest studies of the correlation between serum ferritin and LIC in hemodialysis patients using T2*MRI found a positive correlation between these two variables. Therefore, it was suggested that serum ferritin value above 290 mcg/L should justify further investigation with MRI to rule out iron overload in such cases.^24^,^25^ Though different observation was reported in one EDRD patient with a serum ferritin level of >1000 mcg/L, his LIC measured by T2*MRI was suggestive of only mild iron accumulation liver.^12^ In our case, we also observed that despite the serum ferritin was >1000 mcg/L for several years, the T2*MRI of the heart and liver showed no evidence of iron overload. Our finding could support the idea that serum ferritin might not correlate with LIC and cardiac iron concentration in ESRD patients.

## Conclusion

Despite that, the existing guidelines recommend serum ferritin and transferrin saturation for monitoring secondary iron overload in CKD patients; the evidence for the correlation between serum ferritin and LIC measured by MRI is still scarce in CKD patients. Few studies observe a positive correlation between serum iron and LIC in CKD patients and postulate that serum ferritin above 290 mcg/L should indicate significant iron overload and necessitates MRI evaluation. However, our patient had a serum ferritin level of >1000 mcg/L for several years, but a T2*MRI of the heart and liver revealed the absence of iron overload. We think that further studies are necessary to prove this correlation and determine the cutoff level of serum ferritin that requires further evaluation of iron overload by MRI in CKD patients.
